# Comparison of Normal Saline, Hypertonic Saline Albumin and Terlipressin plus Hypertonic Saline Albumin in an Infant Animal Model of Hypovolemic Shock

**DOI:** 10.1371/journal.pone.0121678

**Published:** 2015-03-20

**Authors:** Javier Urbano, Rafael González, Jorge López, María J Solana, José M. Bellón, Marta Botrán, Ana García, Sarah N. Fernández, Jesús López-Herce

**Affiliations:** 1 Pediatric Intensive Care Department, Gregorio Marañon University Hospital, Madrid, Spain, and Instituto de Investigación Sanitaria Hospital Gregorio Marañón (IiSGM), Research Network on Maternal and Child Health and Development (RedSAMID II), Spanish Health Institute Carlos III, Madrid, Spain; 2 Health Research Fund, Spanish Health Institute Carlos III, Madrid, Spain; 3 Preventive and Quality Control Service, Instituto de Investigación Sanitaria del Hospital Gregorio Marañón Madrid (IiSGM), Madrid, Spain; Erasmus Medical Centre, NETHERLANDS

## Abstract

**Introduction:**

In series of cases and animal models suffering hemorrhagic shock, the use of vasopressors has shown potential benefits regarding hemodynamics and tissue perfusion. Terlipressin is an analogue of vasopressin with a longer half-life that can be administered by bolus injection. We have previously observed that hypertonic albumin improves resuscitation following controlled hemorrhage in piglets. The aim of the present study was to analyze whether the treatment with the combination of terlipressin and hypertonic albumin can produce better hemodynamic and tissular perfusion parameters than normal saline or hypertonic albumin alone at early stages of hemorrhagic shock in an infant animal model.

**Methods:**

Experimental, randomized animal study including 39 2-to-3-month-old piglets. Thirty minutes after controlled 30 ml/kg bleed, pigs were randomized to receive either normal saline (NS) 30 ml/kg (n = 13), 5% albumin plus 3% hypertonic saline (AHS) 15 ml/kg (n = 13) or single bolus of terlipressin 15 μg/kg i.v. plus 5% albumin plus 3% hypertonic saline 15 ml/kg (TAHS) (n = 13) over 30 minutes. Global hemodynamic and tissular perfusion parameters were compared.

**Results:**

After controlled bleed a significant decrease of blood pressure, cardiac index, central venous saturation, carotid and peripheral blood flow, brain saturation and an increase of heart rate, gastric PCO_2_ and lactate was observed. After treatment no significant differences in most hemodynamic (cardiac index, mean arterial pressure) and perfusion parameters (lactate, gastric PCO_2_, brain saturation, cutaneous blood flow) were observed between the three therapeutic groups. AHS and TAHS produced higher increase in stroke volume index and carotid blood flow than NS.

**Conclusions:**

In this pediatric animal model of hypovolemic shock, albumin plus hypertonic saline with or without terlipressin achieved similar hemodynamics and perfusion parameters than twice the volume of NS. Addition of terlipressin did not produce better results than AHS.

## Introduction

Hemorrhagic shock is one of the main causes of morbidity and mortality in children with trauma [1]. Initial treatment attempts to control bleeding and maintain hemodynamic parameters with fluid expansion. However, the best type and the amount of fluid to be administered are not well established. International guidelines on the treatment of hypovolemic shock in children recommend administering a fast bolus of 20 ml/kg of isotonic crystalloid when the peripheral perfusion is inadequate even if the blood pressure is normal and repeating with a second bolus of 20 ml/kg when the heart rate, level of consciousness and capillary filling do not improve [2].

Prehospital fluid resuscitation is limited by the difficulty in delivering large volumes of fluid in the field and time delays, increasing the total time of prehospital care. If treatment is not effective, multiple organ failure rapidly develops, with high mortality [3].

Alterations of tissue perfusion and microcirculation are important factors involved in the development of multiple organ failure. Patients with adequate hemodynamic parameters can present with alteration of general microcirculation parameters and develop multiple organ failure. Therefore, the treatment of hemorrhagic shock must attempt to not only restore general hemodynamic parameters but also to maintain tissue perfusion and microcirculation [4].

Overly aggressive crystalloid resuscitation is associated with poor outcome [5]. Excessive fluid administration has been implicated as a causative factor in coagulation disturbances, abdominal compartment syndrome, pulmonary and cardiac dysfunction and gastrointestinal ileus [6,7]. Experimental evidence also suggests that increases in extracellular volume and changes in cellular osmolarity results in cytosolic acidification, disturbances of cellular phosphorylation, and an increased production of proinflammatory mediators [8].

Previous studies have shown that small volume resuscitation with hypertonic solutions and hypertonic hyperoncotic solutions are superior to saline solutions and colloids achieving a better modulation of the immune system in patients [9,10] and an improvement in survival in animal models [11]. In order to prolong the expansor effect, hypertonic fluids have been combined with colloid solutions, aiming to increase the oncotic pressure of plasma and to retain mobilized fluid within the intravascular space for a longer period of time [12]. Several adult and infant animal studies [11–13] and a meta-analysis in adult trauma patients [14] have found that hypertonic solutions with colloids are more effective than saline in the treatment of hemorrhagic shock. In children suffering from dengue shock syndrome, hypertonic sodium lactate solution achieved similar hemodynamic recovery and survival than Ringer lactate, with less volume and endothelial cell inflammation [15]. However, these benefits have not been observed in the largest clinical trial on the out-of-hospital use of hypertonic versus isotonic resuscitation fluids in adult trauma patients with hemorrhagic shock [16]. The trial was stopped prematurely after the inclusion of 850 patients because of a tendency toward a lower survival rate in patients treated with hypertonic fluids which had not received blood products at early stages. No differences were found in 28 day survival rate.

The use of vasopressors may be beneficial in the management of fluid-resistant uncontrolled hemorrhagic shock, as suggested in recent international guidelines [17]. Terlipressin (TP) is a synthetic analogue of arginine vasopressin (AVP), an endogenous neurohypophysial hormone that enhances vasoconstriction by suppressing the endothelial release of nitric oxide (NO). It has a longer half-life and can be administered by bolus injection. Vasopressin or TP improved hemodynamic status or survival from controlled and uncontrolled hemorrhagic shock in experimental animal models [18–23], and survival rates in a recent meta-analysis of uncontrolled hemorrhage randomized animal trials [24]. There are only series of cases in adults with hemorrhagic shock refractory to fluids and catecholamines that improved with AVP [25].

There is limited experience with hypertonic colloid solutions in children, and no experience with hypertonic albumin solutions. Few studies have examined the efficacy of hypertonic isooncotic solutions in infant animal models of hemorrhagic shock, or have compared the results with saline or colloids [13]. On the other hand, to our knowledge no previous experiences in children or infant animal models have analyzed the effect of AVP or TP in hemorrhagic shock. Clinical experience in children is scarce and controversial. Reports and series of cases include a wide range of age of treated patients for catecholamine-refractory septic shock, from preterm infants to adolescents [26]. The majority of responses observed include rapid improvement in hemodynamic parameters, decrese in serum lactate and dose of inotropes. Conversely, a trend toward higher mortality was observed in a randomized trial comparing low dose of AVP with placebo as an adjunct therapy in pediatric vasodilatory shock [27].

Our hypothesis is that the combination of TP plus hypertonic albumin would reach better hemodynamic and perfusion parameters than isotonic crystalloid (normal saline) or hypertonic albumin in an infant animal model of hemorrhagic shock. Vasopressin and TP have been mostly used as rescue therapies in severe uncontrolled hemorrhagic shock. In this study, our aim was to analyze the potential benefit of the use of TP associated with hypertonic colloid fluid in early stages of hemorrhagic shock, but not necessarily severe hemorrhage, in order to know if this practice should be recommended as a general treatment of hemorrhagic shock in children.

## Methods

### Ethic Statement

The experimental protocol was approved by the Institutional Ethics Committee for Animal Research of the Gregorio Marañon University Hospital, Madrid, Spain (permit number: 12/0013). European and Spanish guidelines for ethical conduct in the care and use of experimental animals were applied throughout the study. The experiments were performed in the Department of Experimental Medicine and Surgery, Gregorio Marañon University Hospital, Madrid, Spain. All efforts were made to minimize suffering.

### Animals

Forty five healthy 2-to-3-month-old (9.9 ± 2.1 kg) Maryland pigs participated in the study. Animals were fed a standard swine diet and observed for a minimum of 2 days to ensure good health. Food was withdrawn the night before the procedures, although water was provided ad libitum.

### Surgical procedures

#### Anesthesia and Instrumentation

After premedication with intramuscular ketamine (15 mg/kg) and atropine (0.02 mg/kg) and monitoring, anesthesia was induced by intravenous boluses of propofol (5 mg/kg), fentanyl (5 μg/kg) and atracurium (0.5 mg/kg). Following tracheal intubation, ventilation was maintained using a mechanical ventilator Servo 900C (Siemens, Munich, Germany) with a respiratory rate of 20 breaths per minute, tidal volume of 10 mL/kg, FiO_2_ of 40%, and positive end-expiratory pressure of 3 cmH_2_O. Ventilation was adjusted to achieve PaCO_2_ between 35 and 45 mmHg. These parameters were adjusted at baseline and maintained constant throughout the experiment despite the changes in the arterial blood gases after hemorrhage. Sedation and muscle relaxation (propofol 10 mg/kg/h, fentanyl 10 μg/kg/h, and atracurium 2 mg/kg/h, by continuous infusion, total amount 1 mL/kg/h) were maintained during the course of the procedure. Core temperature was kept between 36°C and 38°C using a heating blanket with continuous temperature monitoring.

#### Cannulation of vessels

Internal carotid, external jugular, and femoral vessels were identified after exposure via cut-down technique. A 4F catheter was inserted into the femoral artery to measure the blood pressure and cardiac output using a femoral arterial thermodilution system (PiCCO, Pulsion Medical Systems, Munich, Germany). A 4 F catheter was inserted into the external jugular vein and a 5.5 F catheter was inserted in the femoral vein to measure central venous pressure (CVP), and perform blood extraction and volume infusion.

### Hemodynamic and perfusion monitoring

Monitoring included ECG, peripheral oxygen saturation, respiratory volumes and pressures, and FiO_2_ and EtCO_2_ measured by means of a spirometer connected to the endotracheal tube and an S5 monitor (Datex Ohmeda S5, Madison, Wisconsin, USA).

Brain tissue oxygenation index (bTOI) and splanchnic tissue oxygenation index (aTOI) were monitored by near-infrared spectroscopy (NIRS) (INVOS Cerebral Oximeter monitor, Somanetics, Troy, Michigan, USA) with sensors positioned on the skin of forehead and anterior abdominal wall (subhepatic region), respectively.

Internal carotid arterial blood flow (CaBF) was measured by a specific sensor connected to a flow monitor (Transonic Systems Inc, Ithaca, New York, USA).

To measure gastric intramucosal pH (pHi), a 7F tonometric catheter (TRIP, Tonometrics Division, Instrumentarium Corp, Helsinki, Finland) was passed into the stomach and connected to an S5 Monitor (Datex-Ohmeda Madison, USA). No histamine type 2 (H_2_) receptor blockers were administered. The tonometer balloon was filled with air, and automatic sampling was performed every 10 minutes.

A Laser Doppler sensor BLF21A (Transonic Systems Inc, Ithaca, Nueva York, USA), was placed over the skin of the anterior abdominal wall, and was used for continuous measurement of cutaneous tissular blood flow (CuTBF).

The following parameters were recorded at baseline and every 30 minutes during the experiment: blood temperature, inspiratory tidal volume, EtCO_2_, cardiac rhythm, heart rate (HR), systolic and diastolic blood pressures, mean arterial blood pressure (MAP), CVP, cardiac index (CI), global end diastolic volume index (GEDVI), stroke volume index (SVI), left ventricular contractility (Dt/Dpmax), systemic vascular resistance index (SVRI), extravascular lung water index (ELWI), pressure pulse variation (PPV), peripheral hemoglobin saturation, central venous saturation (ScvO_2_), cerebral (bTOI) and splanchnic (aTOI) tissue oxygenation index by NIRS, intramucosal pH (pHi), gastric intramucosal pCO_2_ (PgCO_2_), CaBF and CuTBF.

Cardiac output (CO) was taken as the average of 2 consecutive measurements using 5 mL boluses of 0.9% NS at a temperature below 8°C administered via the central venous catheter.

### Blood draws

Arterial and venous blood gas profiles and lactate concentration were determined every 30 minutes by drawing 0.3 mL of blood from the femoral vessels. Blood gases were analyzed using the GEM Premier 3000 blood gas analyzer (Instrumentation Laboratory, Lexington, Kentucky, USA). An additional 4 mL of blood was drawn from the femoral artery at baseline, end of hemorrhage and end of the observation period. Standard complete blood counts, coagulation studies and biochemistry (including electrolytes, total protein, albumin, aspartate aminotransferase, alanine aminotransferase, urea, creatinine, and troponin) were performed.

After each blood draw, lines were flushed with 0.5 mL of heparin 100 IU/mL.

### Treatment protocol

**Fig 1:**
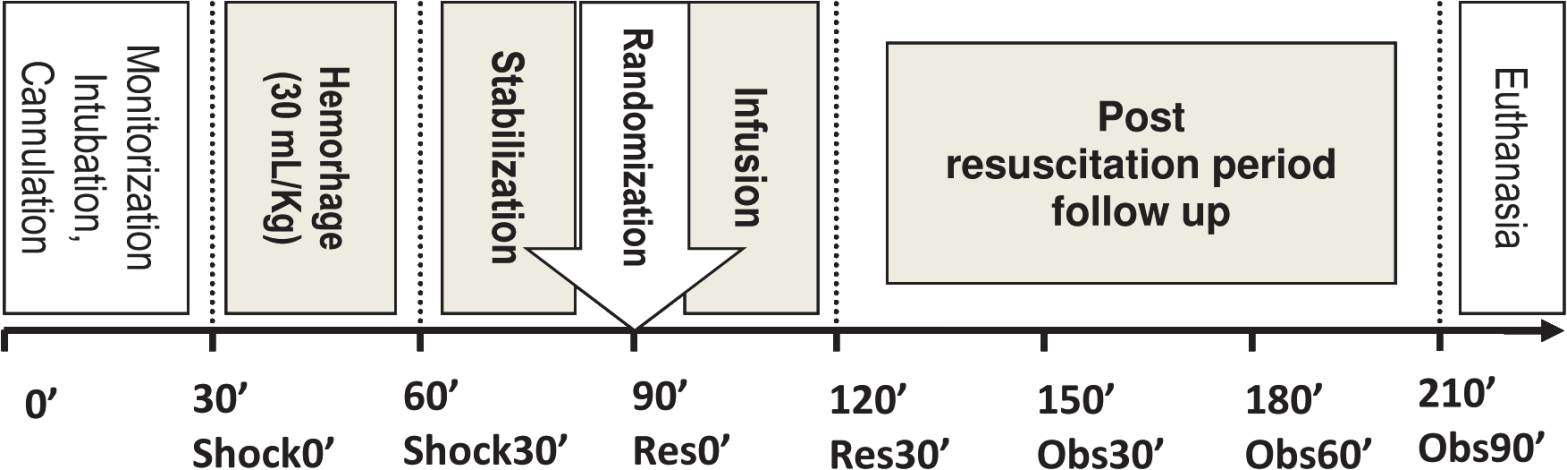
Timeline for the experiment.

The experiment was divided into five different periods (Fig. 1): Cannulation, controlled haemorrhage, stabilization, infusion, and follow up for 90 minutes after infusion. Following surgical preparation, the animals were allowed to stabilize for 30 minutes. Once a steady state was achieved and baseline (t0’) data were gathered, hypovolemic shock was induced by the withdrawal of 30 mL/kg of blood over 30 minutes (shock30’). This model has been shown to successfully discriminate between different resuscitation fluids as has been previously reported by our group [13]. Three animals were excluded for technical problems during cannulation and 3 died during the hemorrhage period prior to the initiation of the infusion. After a 30-minute stabilization period (Res0’) mimicking the time required for first aid team arrival, hemorrhage control and intravenous line placement on the pre-hospital setting, 39 animals were randomized to receive either normal saline (NS) 30 mL/kg (n = 13), 5% albumin plus 3% hypertonic saline (AHS) 15 mL/kg (n = 13), or simultaneous single bolus of terlipressin (Glypressin, Ferring Pharmaceuticals, Saint-Prex, Switzerland) 15 μg/kg iv plus 5% albumin 3% hypertonic saline 15 ml/kg (TAHS) (n = 13) over 30 minutes. The amount of NS and AHS was determined based on our previous study [13]. The dose of TP was determined based on a previous study [20]. The randomization scheme was generated by a computer program in the web site http://www.randomization.com. Investigators were not blind to the treatment.

The fluid was administered over a period of 30 minutes (*Res30’*). During the next 90 minutes, no resuscitation effort was made, and parameters were recorded each 30 minutes (*Obs30’*, *Obs60’* and *Obs90’*, respectively). AHS was prepared by the addition of 22 mL of 20% NaCl plus 50 mL of 20% albumin to each 128 mL of 0.9% NS. Terlipressin was prepared to a final concentration of 20 μg of drug per mL of 5% glucose, which represented a volume load of 0.75 mL/kg.

On completion of the experiment, all successfully resuscitated animals were sacrificed by the administration of sedative overdose (fentanyl and propofol) and the intravenous injection of potassium chloride.

### Statistical analysis

The primary endpoint was Cardiac Index. Based on our previous study [13], we performed a power analysis which calculated 11 pigs per group. The statistical analysis was performed using the SPSS statistical package, version 20.0 (SPSS Inc, Chicago, USA). The Pearson’s Chi-squared test and the Fisher’s exact test were used for qualitative variables analysis. Multiple analysis of variance (MANOVA) for repeated measures was used to study the changes in the parameters over the course of the experiment and between-group comparison. The Bonferroni test was used to adjust multiple comparisons. Data are shown as means and standard deviations, otherwise specified. P values less than 0.05 were considered significant.

Normal distribution of variables was tested with the Kolmogorov-Smirnov test. The percentage of variation of each parameter between the value after shock before the beginning of the treatment and after volume expansion was compared using Mann—Whitney U test for non-normal distributed variables.

## Results

**Fig 2:**
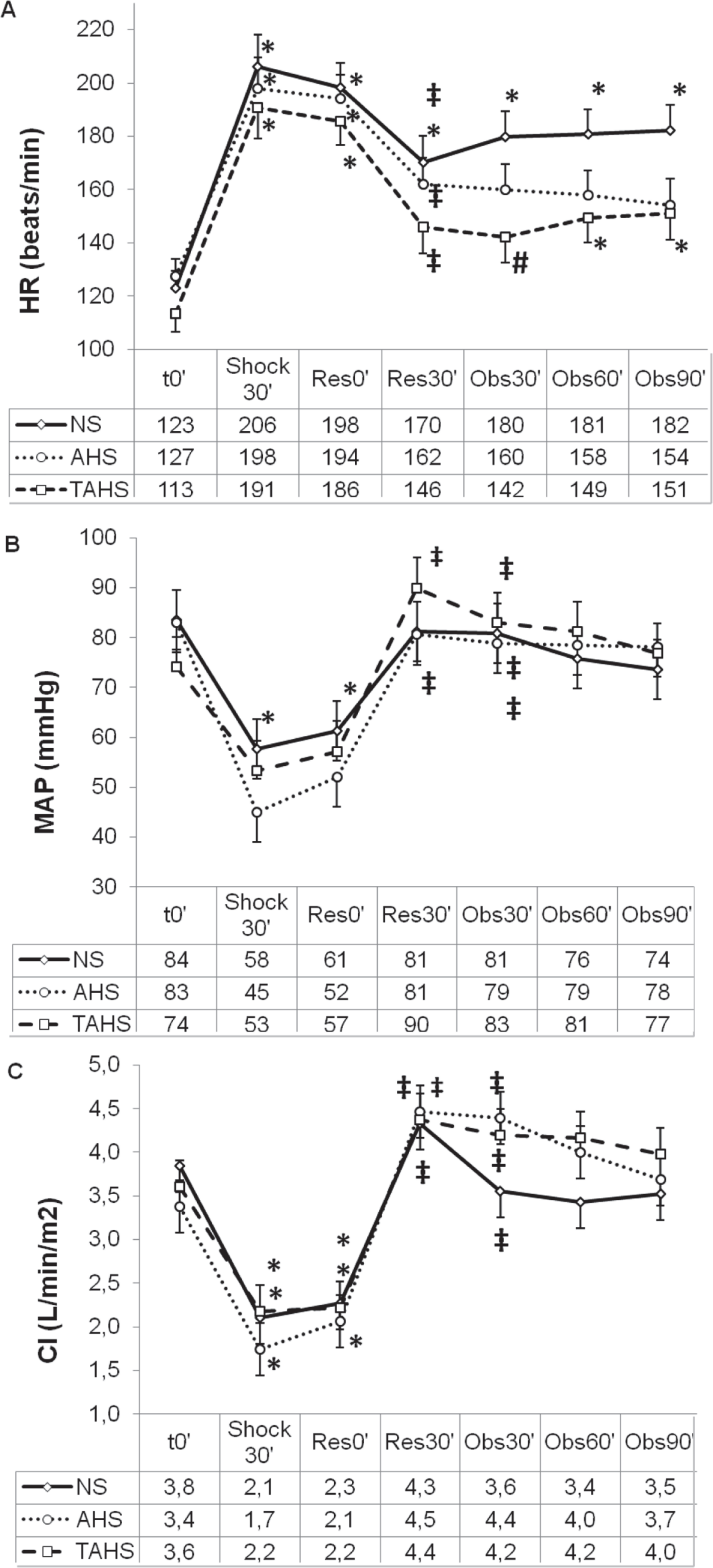
Evolution of heart rate (A), mean arterial pressure (B) and cardiac index (C) at baseline (t0’); and throughout the course of the experiment: end of controlled bleeding (Shock30’); beginning of infusion, 30 min after the end of controlled bleeding (Res0’); end of infusion (Res30’); follow up 30 min after the end of the infusion (Obs30’); follow up 60 min after the end of infusion (Obs60’); follow up 90 min after the end of infusion (Obs90’). (*) Significant difference (p < 0.05) from baseline, same group. (‡) p < 0.05 from hemorrhage, same group. (#) p < 0.05 from group NS.

**Fig 3:**
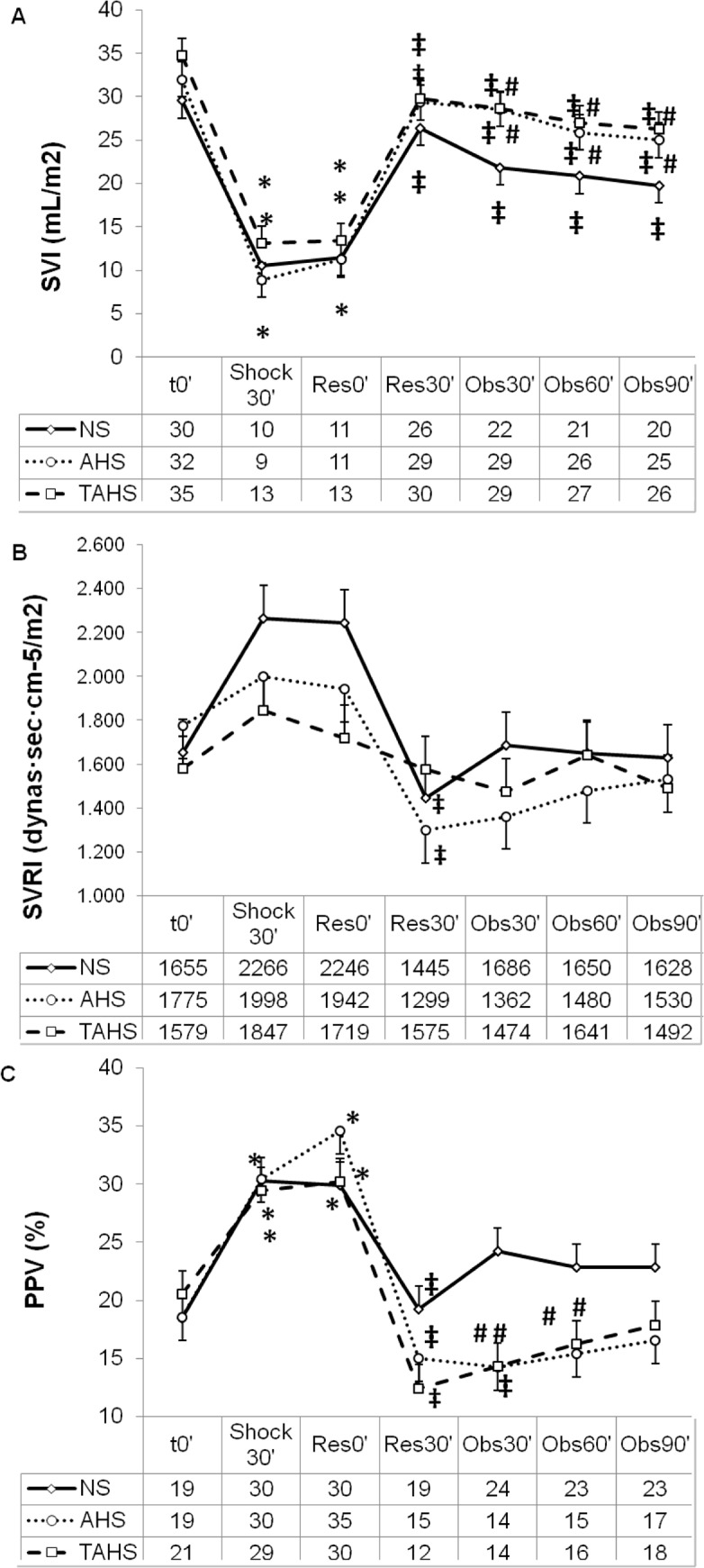
Evolution of stroke volume index (A), systemic vascular resistance index (B) and pulse pressure variation (C) at baseline (t0’); and throughout the course of the experiment: end of controlled bleeding (Shock30’); beginning of infusion, 30 min after the end of controlled bleeding (Res0’); end of infusion (Res30’); follow up 30 min after the end of the infusion (Obs30’); follow up 60 min after the end of infusion (Obs60’); follow up 90 min after the end of infusion (Obs90’). (*) Significant difference (p < 0.05) from baseline, same group. (‡) p < 0.05 from hemorrhage, same group. (#) p < 0.05 from group NS.

**Fig 4:**
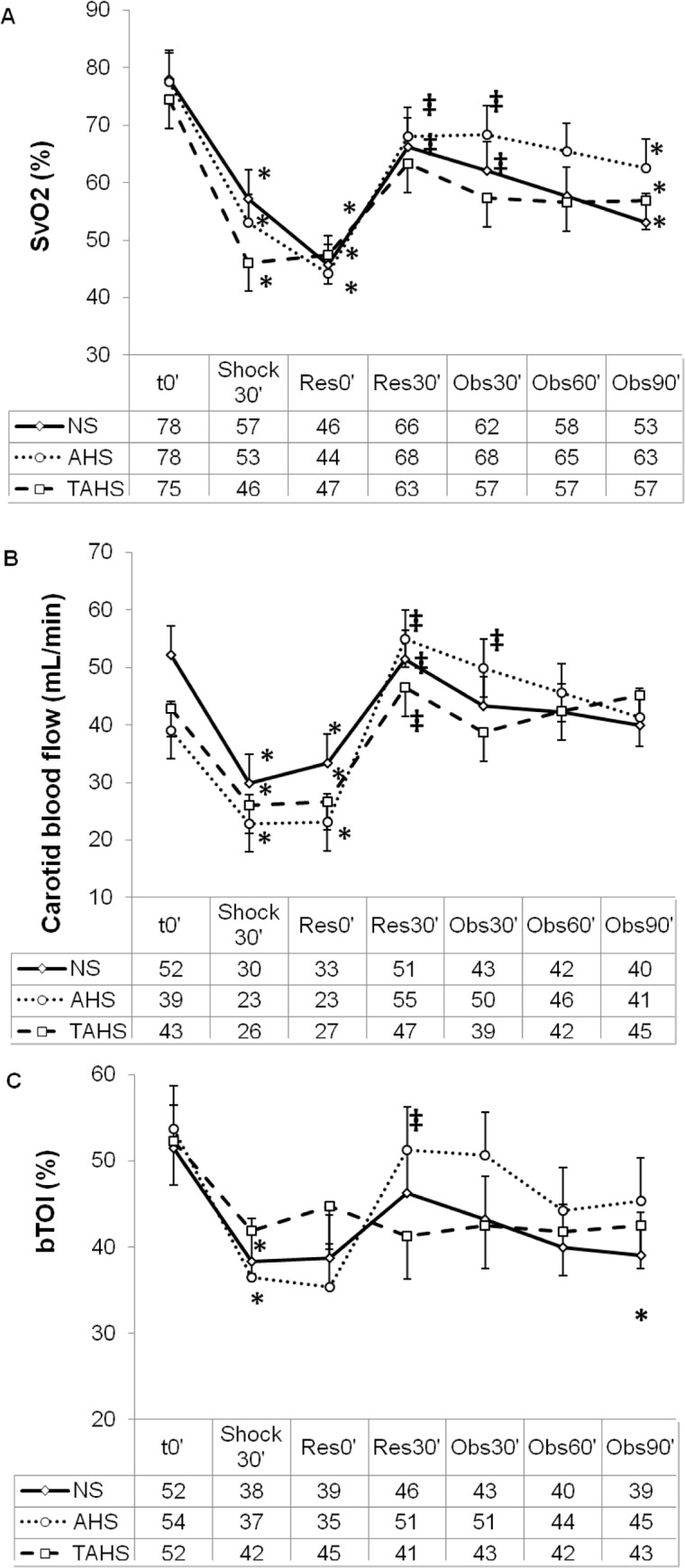
Evolution of central venous blood oxygen saturation (A), carotid blood flow (B) and brain tissue oxygenation index (C) at baseline (t0’); and throughout the course of the experiment: end of controlled bleeding (Shock30’); beginning of infusion, 30 min after the end of controlled bleeding (Res0’); end of infusion (Res30’); follow up 30 min after the end of the infusion (Obs30’); follow up 60 min after the end of infusion (Obs60’); follow up 90 min after the end of infusion (Obs90’). (*) Significant difference (p < 0.05) from baseline, same group. (‡) p < 0.05 from hemorrhage, same group. (#) p < 0.05 from group NS.

**Fig 5:**
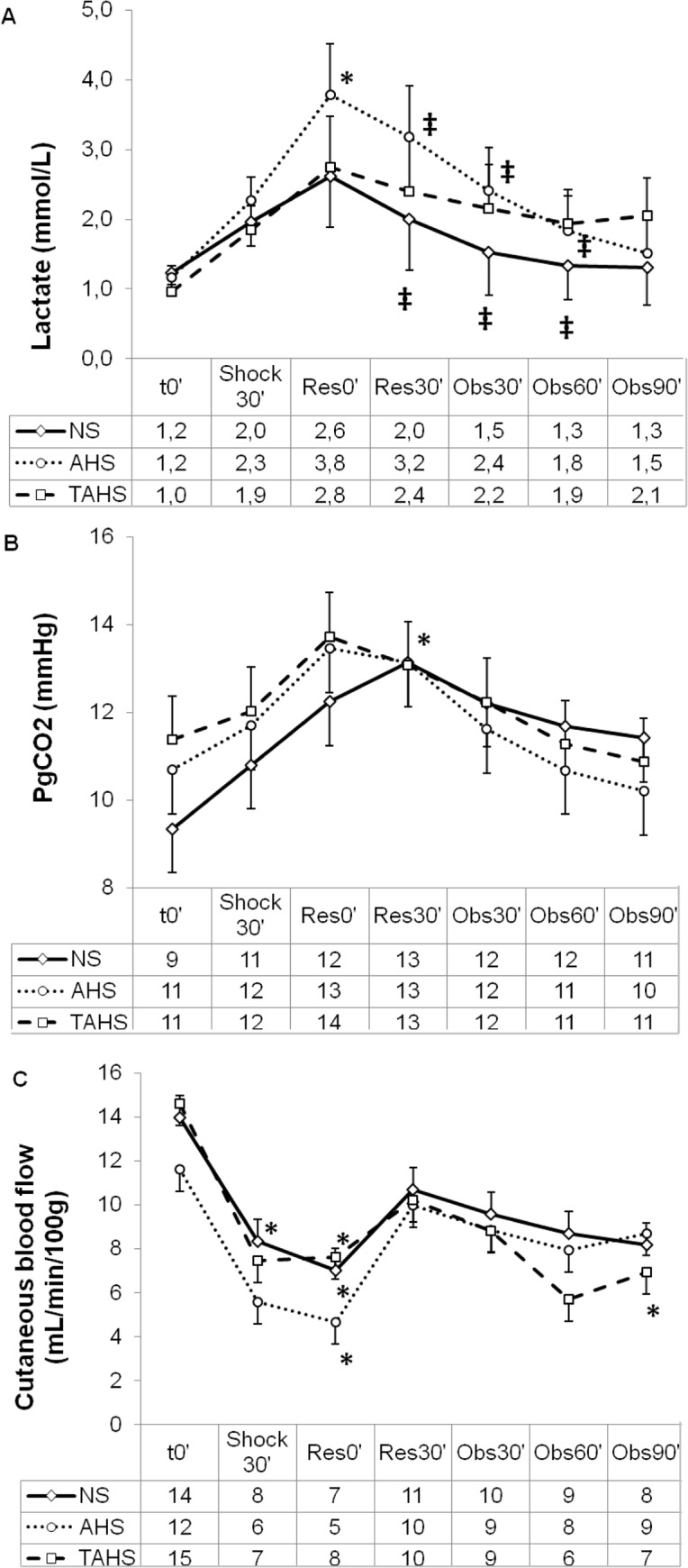
Evolution of arterial blood lactate (A), gastric intramucosal pCO_2_ (B) and cutaneous blood flow (C) at baseline (t0’); and throughout the course of the experiment: end of controlled bleeding (Shock30’); beginning of infusion, 30 min after the end of controlled bleeding (Res0’); end of infusion (Res30’); follow up 30 min after the end of the infusion (Obs30’); follow up 60 min after the end of infusion (Obs60’); follow up 90 min after the end of infusion (Obs90’). (*) Significant difference (p < 0.05) from baseline, same group. (‡) p < 0.05 from hemorrhage, same group. (#) p < 0.05 from group NS.

The study groups did not differ with respect to weight or baseline hemodynamic, perfusion, and laboratory parameters (Table 1, Figs. 2 to 5).

**Table 1:** Comparison of analytic parameters between the three treatment groups at baseline, end of hemorrhage and end of experiment.

		Baseline		Shock 30'		Obs 90'	
Parameter		Mean (SD)	P	Mean (SD)	P	Mean (SD)	P
**Hemoglobin** (g/dL)	NS	8.2 (0.6)	.339	8.5 (0.4)	.769	6.5 (0.4)	.009
	AHS	8.7 (0.6)		8.3 (0.4)		5.1 (0.4)	
	TAHS	7.4 (0.6)		8.0 (0.5)		4.6 (0.4)	
**Leucocytes**(x10^9^/L)	NS	13.8 (2.1)	.226	13.5 (1.5)	.154	25.3 (2.6)	.005
	AHS	11.2 (2.3)		11.4 (1.6)		13.8 (2.7)	
	TAHS	8.3 (2.2)		9.0 (1.6)		12.8 (2.7)	
**Na** (mmol/L)	NS	136 (1)	.285	136 (1)	.355	137 (1)	.001
	AHS	135 (1)		134 (1)		148 (1)	
	TAHS	137 (1)		135 (1)		144 (1)	
**Cl** (mmol/L)	NS	100 (1)	.519	101 (1)	.323	105 (1)	.001
	AHS	102 (1)		104 (1)		115 (1)	
	TAHS	101 (1)		102 (1)		112 (1)	
**K** (mmol/L)	NS	3.6 (0.1)	.854	4.9 (0.4)	.478	4.4 (0.2)	.374
	AHS	3.5 (0.1)		5.4 (0.4)		4.3 (0.2)	
	TAHS	3.7 (0.1)		4.8 (0.4)		4.7 (0.2)	
**Arterial pH**	NS	7.46 (0.01)	.995	7.44 (0.01)	.911	7.39 (0.02)	.946
	AHS	7.45 (0.01)		7.42 (0.01)		7.39 (0.02)	
	TAHS	7.45 (0.01)		7.43 (0.02)		7.40 (0.01)	
**EtCO_2_ (mmHg)**	NS	37.5 (1.0)	.986	35.3 (1.6)	.649	37.8 (1.6)	.851
	AHS	37.5 (1.0)		33.4 (1.6)		39.1 (1.6)	
	TAHS	37.7 (1.0)		35.3 (1.6)		38.6(1.6)	
**Temp**. (°C)	NS	37.6 (0.4)	.965	38.0 (0.4)	.900	38.2 (0.3)	.990
	AHS	37.6 (0.4)		38.2 (0.4)		38.2 (0.3)	
	TAHS	37.7 (0.4)		38.3 (0.3)		38.3 (0.3)	
**Creatinine** (mg/dL)	NS	0.5 (0.0)	.543	0.5 (0.0)	.304	0.5 (0.0)	.597
	AHS	0.4 (0.0)		0.5 (0.0)		0.6 (0.0)	
	TAHS	0.4 (0.0)		0.5 (0.0)		0.6 (0.0)	
**Urea** (mg/dL)	NS	30.4 (3.3)	.322	34.6 (3.5)	.255	38.5 (3.5)	.231
	AHS	31.4 (3.4)		36.1 (3.7)		40.3 (3.6)	
	TAHS	24.7 (3.2)		28.0 (3.5)		32.0 (3.5)	
**AST** (IU/L)	NS	34.4 (5.2)	.089	40.2 (10.3)	.333	54.8 (26.6)	.169
	AHS	49.6 (5.2)		55.5 (10.3)		108.5 (26.6)	
	TAHS	35.4 (5.2)		33.9 (10.3)		38.2 (26.6)	
**ALT** (IU/L)	NS	27.8 (2.3)	.151	28.0 (2.4)	.550	24.4 (2.8)	.621
	AHS	31.5 (2.3)		28.6 (2.4)		26.6 (2.8)	
	TAHS	34.3 (2.3)		31.5 (2.4)		22.9 (2.8)	

Shock 30’: end of controlled bleeding before beginning of infusion; Obs90’: 90 minutes after the end of infusion. NS: normal saline; AHS: albumin plus hypertonic saline; TAHS: terlipressin plus albumin hypertonic saline.

### Response to acute hypovolaemia

Following volume withdrawal, the animals presented deep hemodynamic changes regarding HR, MAP, CI, IVS, GEDVI and CaBF (Figs. 2, 3, 4 and S1 Fig.). No statistically significant variations were detected in CVP, SVRI, dPmax and ELWI values (Fig. 3 and S1 Fig.). Systemic perfusion parameters such as lactate, PgCO_2_ and pHi increased significantly, while ScvO_2_, CuTBF and bTOI decreased (Figs. 4 and 5). Splanchnic oxygenation (aTOI) decreased, although only significantly in the NS group (S2 Fig.). Other parameters, including core temperature, EtCO_2_, electrolytes, kidney and liver function parameters, PaO_2_, PaCO_2_, and arterial oxygen saturation remained stable, with no statistically significant differences throughout the course of the experiment (data not shown). There were no significant differences between groups regarding changes in hemodynamic, respiratory and perfusion parameters or in blood-gas profiles with hypovolaemia (Figs. 2 to 5).

### Response to volume expansion and treatment

After fluid expansion a diminution of HR, SVRI, PPV and lactate, and an increase of MAP, CI, SVI, GEDVI, dPmax, ScvO_2_, bTOI and CaBF was observed (Figs. 2 to 5, and S1 Fig.).

#### Comparison between treatment groups

There were no significant differences in most of the hemodynamic and perfusion parameters between the three treatment groups at 30, 60 and 90 minutes after expansion.

Thirty minutes after expansion the HR and PPV were lower and SVI was higher in the AHS and TAHS groups than in the NS group (Fig. 2). Sixty minutes after fluid expansion AHS and TAHS groups showed higher values of SVI and lower PPV than NS group (Fig. 3). Thirty minutes after expansion GEDVI progressively decreased to values lower than baseline in NS group (S1 Fig.). CuTBF significantly increased at 30 and 60 minutes after expansion in NS and AHS groups but not in TAHS (Fig. 5).

Ninety minutes after fluid expansion, NS group had a significantly higher hemoglobin, and leucocytes, and lower Na and Cl than in the other groups (Table 1). A secondary analysis has been performed excluding the NS group in order to improve the ability to detect differences between AHS and TAHS groups without employing a Bonferroni correction, since comparisons between NS and AHS in the same animal model have been previously explored (13). Thirty minutes after fluid expansion, ScvO_2_ and CaBF were higher in the AHS group than in the TAHS (p = 0.030 and p = 0.036, respectively).

#### Initial response to treatment

Thirty minutes after fluid expansion AHS group achieved a greater increase in CI, SVI and a greater decrease in PPV than NS group; as well as a greater increase of bTOI, CaBF, and a greater decrease of lactate levels than the other groups in comparison with the beginning of the fluid expansion (Table 2). In TAHS group the diminution of lactate 30 minutes after expansion was lower than in the AHS group (Fig. 4), and the increase of CuTBF was lower than the other groups. Sixty minutes after expansion AHS and TAHS group showed greater increase in CaBF and greater decrease in PPV than NS group. AHS group showed greater increase in SVI than NS group (Fig. 5). 60 minutes after expansion TAHS group showed lower diminution of lactate in comparison with the beginning of the treatment than the other groups (Fig. 5).

**Table 2:** Variations of hemodynamic and perfusion parameters at 30 and 60 minutes after the end of the infusion.

Variation		Shock30’-Obs30’	Shock30’-Obs60’
Parameter	Group	Δ% (SD)	Δ% (SD)
**CI**	NS	64 (37)	57 (25)
	AHS	137 (110) ^a^	112 (85)
	TAHS	111 (90)	109 (90)
**SVI**	NS	101 (71)	92 (57)
	AHS	182 (89) ^a^	149 (67) ^a^
	TAHS	146 (103)	139 (110)
**GEDVI**	NS	40 (74)	30 (40)
	AHS	50 (29)	38 (19)
	TAHS	49 (19)	45 (21)
**PPV**	NS	−19 (14)	−22 (17)
	AHS	−58 (11) ^a^	−54 (17) ^a^
	TAHS	−48(15) ^a^	−42 (15) ^a^
**Carotid blood flow**	NS	32 (26)	30 (27)
	AHS	204 (230) ^a^ ^,^ ^b^	169 (177) ^a^ ^,^ ^b^
	TAHS	37 (46)	49 (24)
**bTOI**	NS	15 (22)	5 (23)
	AHS	68 (78) ^a^ ^,^ ^b^	52 (81) ^a^ ^,^ ^b^
	TAHS	2 (21)	2 (17)
**Cutaneous blood flow**	NS	41 (54)	35 (41)
	AHS	133(174) ^a^ ^,^ ^b^	111 (170) ^b^
	TAHS	−2 (44) ^a^	−16 (30) ^a^
**Lactate**	NS	−40 (8)	−46 (13)
	AHS	−43 (16) ^b^	−55 (12) ^b^
	TAHS	−21 (20)	− 27 (19) ^a^
**PgCO**_**2**_	NS	2 (15)	−2 (18)
	AHS	−12 (11)	− 18 (11)
	TAHS	−6 (17)	−12 (19)
**ScvO**_**2**_	NS	49 (55)	33 (29)
	AHS	78 (67)	71 (69)
	TAHS	33 (55)	28 (41)

Shock30’: end of controlled bleeding before beginning of infusion; Obs30’: 30 minutes after the end of infusion. Obs60’: 60 minutes after the end of infusion. NS: Normal saline; AHS: Albumin plus hypertonic saline; TAHS: Terlipressin plus albumin hypertonic saline. CI: Cardiac index; SVI: Stroke volume index; GEDVI: Global end diastolic index; PPV: Pulse pressure variation; bTOI: Brain tissue oxygenation index; PgCO_2_: Gastric intramucosal pCO_2_; ScvO_2_: Central venous blood oxygen saturation. Δ% (SD): variation (in percentage) respect to the previous moment.

^a^ p<0.05 respect to NS.

^b^ p<0.05 respect to TAHS.

## Discussion

This is the first experimental study to compare the effects of NS, AHS and TAHS in a pediatric animal model of hypovolemic hemorrhagic shock, and to analyze their effect on hemodynamic and tissue perfusion parameters.

Hypovolemic hemorrhagic shock produced marked changes in hemodynamic parameters and perfusion parameters as observed in previous studies [13,28].

The main results of this study are that the infusion of hypertonic albumin, with or without TP, produced a similar response to the infusion of twice the volume of isotonic fluid, and that the addition of a single bolus of TP at the beginning of the fluid resuscitation did not present any benefits in hemodynamic or perfusion parameters. These results suggest that the TP bolus has provoked a decrease in CaBF and bTOI, CuTBF, ScvO_2_ and clearance of serum lactate.

### Hemodynamic response

The three fluid treatments obtained similar global hemodynamic values after expansion. However, NS needed twice as much volume. On the other hand, AHS and TAHS produced a greater and longer volume expansion than NS (with significant differences in SVI and PPV, and the absence of a progressive fall after volume expansion in GEDVI as observed in NS group). This is probably secondary to the capacity of albumin to increase blood volume more and to remain longer within the intravascular compartment than normal saline [29]. Although AHS and TAHS showed higher and longer increase in CI than NS, the differences were not significant, probably because these groups showed lower HR than NS. So it seems that AHS and TAHS maintain CI with higher stroke volume and lower heart rate than NS (Fig. 2).

Hypertonic fluids produce an immediate mobilization of intracellular water into the intravascular extracellular space, increasing hydrostatic pressure and intracellular osmolality. Hypertonic fluids achieve a greater increase in preload, diuresis and an early fall in afterload than isotonic fluids [11,14]. However, only a previous experimental study analyzed resuscitation with a combination of 5% albumin plus 3% hypertonic saline [13].

Our study confirmed that AHS produces greater and longer volume expansion and this fact could permit a faster restoration of the hemodynamic parameters. According to the data in our previous study comparing NS with HS and AHS, half of the volume of HS or AHS achieved similar hemodynamic and perfusion endpoints than NS despite differences in hemoglobin concentration [13].

Arginine vasopressin (AVP) is a vasoconstrictor that suppresses nitric oxide production and may contribute to the compensatory cardiovascular mechanisms aimed at preserving blood flow to vital organs. It has been shown that even after severe prolonged hemorrhagic hypotension, blood pressure remains responsive to exogenous AVP [21]. However, few studies have analyze the effect of terlipressin. Interestingly, in a rat model study comparing the effects of both treatments, terlipressin achieved more prolonged time of restored mean arterial pressure than AVP. Both treatments were associated to a lower inflammatory cytokine profile and lung histopathology than lactated ringer [22]. In children, TP has been used in catecholamine refractory hypotensive septic shock [26,30].

In a research review about the use of AVP in hemorrhagic shock, the authors recommend to study the benefits of administering a low-dose AVP infusion early in resuscitation, previous to the onset of the decompensatory phase of hemorrhagic shock [31]. Several experimental studies have found that AVP could improve hemodynamics and survival in experimental models of hemorrhagic shock and in some adult patients with hemorrhagic shock refractory to fluids [24,25]. A recently published meta-analysis included 15 randomized animal trials evaluating the benefits of AVP or TP in terms of survival of hemorrhagic shock [24]. Only one study used TP [18]. The results showed an odds ratio of 0.09, with a 95% CI (0.05–0.15) favoring the use of AVP or TP. This benefit was observable when comparing AVP or TP with other vasopressors, fluid resuscitation with crystalloids and colloids (both iso- and hypertonic), or whole blood. Eleven out of the 15 studies included used an uncontrolled hemorrhagic shock model, with severe hypotension (MAP lower than 30–40 mmHg in adult animals) and metabolic acidosis; nine of them used a liver injury or penetrating abdominal trauma [24]. The cases reported in humans included severe hemorrhagic shock resistant to adequate fluid resuscitation and catecholamine infusion [25]. In contrast, in a controlled hemorrhagic shock model study comparing the use of AVP plus fluid resuscitation versus fluids alone, the authors observed lower CI and higher lactate and metabolic acidosis in the AVP group, despite needing a lower volume to achieve a determined MAP target [21]. Moreover, the infusion of AVP to hemodynamically stable pigs lowered the cerebral oxygenation index and cerebral blood volume [32].

Our results show that the association of TP and hypertonic albumin did not achieve better hemodynamic parameters than hypertonic albumin alone. This may have several explanations: 1) the infusion of AHS achieved a complete normalization of hemodynamic parameters. Perhaps a smaller volume resuscitation strategy using only 4–5 ml/kg of fluid would show differences. Nevertheless higher volumes (up to 100 ml/kg administered in two hours) associated to TP have been used in previous studies where positive effects have been found [20]. But it is still surprising that TAHS only produced a slightly non significant higher MAP and SVRI than AHS alone. 2) In several previous studies the use of AVP in an otherwise 100% lethal hemorrhage experiments resulted in a 100% survival [23,24]. Therefore, it is possible that the shock induced in the present study was not severe enough to show differences attributable to the use of TP. 3) A paradoxical vasodilatation phenomenon secondary to the use of high doses of TP after hemorrhagic shock has been postulated in two previous studies [19,20]. However, no differences in CI or SVRI have been observed in the previous study. Moreover, despite Devlin et al. postulated this paradoxical effect, significantly less amount of fluids was required to maintain MAP in the group were the highest dose of TP was administered (15 mcg/kg) [20]. 4) The dose of terlipressin was not high enough to cause a difference. A recent study compared the treatment with a higher dose of 50 μg/kg of terlipressin, without fluid expansion, versus 4 ml/kg bolus of lactated ringer in an uncontrolled liver hemorrhage rat model. The terlipressin group reached longer time and higher survival rates, requiring less amount of fluid to maintain MAP, despite no improvement in perfusion parameters was observed [18]. However, similar or even lower doses as the dose administered in the present study, combined with small volume resuscitation in the swine have demonstrated positive effects with a lower risk of adverse effects, mainly ischemia [20,23,33].

### Perfusion parameters

Our results show that AHS produced better results in perfusion parameters than the other two treatments, but there were only significant differences in bTOI, ScvO_2_ and lactate.

Fluid expansion completely normalized hemodynamic and perfusion parameters except central venous saturation. ScvO_2_ has been recommended as an objective parameter of hemodynamic stabilization in hypovolemic shock [34]. The incomplete normalization of ScvO_2_ could be produced by a reduction in oxygen transport due to the loss of red blood cells and haemodilution secondary to treatment, despite the improvement in the hemodynamic situation [35]. Arterial lactate concentration reflects the oxygen debt [36]. In our study, the 3 treatments significantly reduced lactate concentration, but AHS produced a greater decrease of lactate suggesting a better improvement of microcirculation.

It has been suggested that the administration of vasopressors without a previous cardiac preload optimization may lead to a paradoxical decrease in cardiac output and a secondary elevation of lactate levels [19–21]. In our study, simultaneous administration of TP and expansion did not produce these effects. Intramucosal PCO_2_ (PgCO_2_) may be increased both by a reduction in its clearance secondary to decreased blood flow and by an increased production secondary to tissue hypoxia and anaerobic metabolism [37]. In our study the three treatments normalized PgCO_2_ suggesting a normalization of splanchnic perfusion. Near infrared spectroscopy indirectly evaluates brain tissue oxygenation [38]. In our study, brain tissue oxygenation index (bTOI) diminished significantly with hemorrhagic shock and was normalized with the three treatments, but AHS produced a significantly higher increase than the other treatments. A decrease in bTOI after the infusion of AVP as has been previously described in hemodynamically stable pigs [32].

### Secondary effects

Treatment with AHS and TAHS produced a slightly significant increase of Na and Cl but hypernatremia and hyperchloremia were moderate and did not produce metabolic acidosis.

Lower hemoglobin concentration was observed 90’ after the end of the infusion in the groups treated with hypertonic albumin (AHS and TAHS), and a higher WBC count in the group treated with NS, despite the fact that half as much volume was used in comparison with the NS group. This could be explained because colloids and hypertonic fluids are able to shift volume from the interstitial to the intravascular space, resulting in a higher volume expanding power than NS and therefore, more hemodilution. This effect has been observed in previous studies [13,39]. It is well documented that the infusion of crystalloids produces an increased activation of neutrophils, and that the infusion of 5% albumin or hypertonic saline have immunomodulatory effects. The difference in the concentration of leukocytes may be attributed to a difference in volume expansion rather than to this phenomenon [8,29].

Treatment with TAHS produced a significantly lower decrease of CuTBF than the other two treatments, without any signs of peripheral ischemia. However, we administered a single dose of vasopressor in our study. It is necessary to evaluate if a higher incidence of peripheral ischemia will occur with repeated doses or continuous infusions of TP, as previously observed [33].

### Limitations

Our study has several limitations. First, our results would apply only to hypovolemic hemorrhagic shock but not to other types of shock. Second, we only analyzed the initial response to controlled hemorrhagic shock but it could not be the same in a more advanced shock or in an uncontrolled hemorrhagic shock model. Uncontrolled hemorrhage models are more realistic, and the effects of the infusion of certain fluids or vasopressors on the amount of bleeding or the coagulation factors may be different [40]. Third, fixed-volume hemorrhagic shock models are suitable to compare the response to treatment after severe hemorrhage as they allow the development of the physiologic reaction of the animal. However, heterogeneous hemodynamic and perfusion derangements after hemorrhage may be observed. Moreover, this physiologic response could possibly be blunted by the administration of anesthetic drugs. Fourth, the inclusion of additional control groups as TP plus NS, and TP alone could possibly improve the design of the study and the knowledge about the effects of TP. However, this would increase the number of pigs to be included, which could be a concern from an ethical point of view. Therefore, in order to reduce this number, we have selected the most relevant groups, according to the preexisting bibliography and our own results. And fifth, it is not known whether the dose of TP used in the swine can be extrapolated to the child. More studies are needed to properly assess the effect of the administration of TP in different types of shock.

### Conclusions

In conclusion, in this pediatric animal model of hypovolemic shock, controlled acute hemorrhage produced important alterations of systemic and perfusion parameters. Hypertonic albumin and hypertonic albumin plus terlipressin achieved similar hemodynamic and perfusion endpoints with half as much the volume than normal saline, without a significant improvement attributable to the use of terlipressin. Animals treated with albumin plus hypertonic saline presented higher blood volume parameters, a greater increase in brain oxygenation and a greater decrease in lactate. Although this experimental study cannot be directly extended to clinical practice, our results suggest that albumin hypertonic saline (AHS) without a vasopressor could be a more suitable fluid for the initial resuscitation rather than normal saline. Therefore, vasopressors should not be used as an initial treatment in controlled hemorrhagic shock in children. New studies should analyze its efficacy in shock refractory to fluids or uncontrolled hemorrhage.

## Supporting Information

S1 Fig Global end diastolic volume index (A), left ventricle’s contractility (B) and extra vascular lung water index (C) at baseline (t0’); and throughout the course of the experiment: end of controlled bleeding (Shock30’); beginning of infusion, 30 min after the end of controlled bleeding (Res0’); end of infusion (Res30’); follow up 30 min after the end of the infusion (Obs30’); follow up 60 min after the end of infusion (Obs60’); follow up 90 min after the end of infusion (Obs90’). (*) Significant difference (p < 0.05) from baseline, same group. (‡) p < 0.05 from hemorrhage, same group. (#) p < 0.05 from group NS.
(TIF)

S2 Fig Splanchnic oxygenation index (aTOI) at baseline (t0’); and throughout the course of the experiment: end of controlled bleeding (Shock30’); beginning of infusion, 30 min after the end of controlled bleeding (Res0’); end of infusion (Res30’); follow up 30 min after the end of the infusion (Obs30’); follow up 60 min after the end of infusion (Obs60’); follow up 90 min after the end of infusion (Obs90’). (*) Significant difference (p < 0.05) from baseline, same group. (‡) p < 0.05 from hemorrhage, same group. (#) p < 0.05 from group NS.
(TIF)
