# Supplementary figures and images for: Comparison of Normal Saline, Hypertonic Saline Albumin and Terlipressin plus Hypertonic Saline Albumin in an Infant Animal Model of Hypovolemic Shock
Source: PLoS One. 2015 Mar 20;10(3):e0121678. doi: 10.1371/journal.pone.0121678 (PMC4368553; doi:10.1371/journal.pone.0121678)

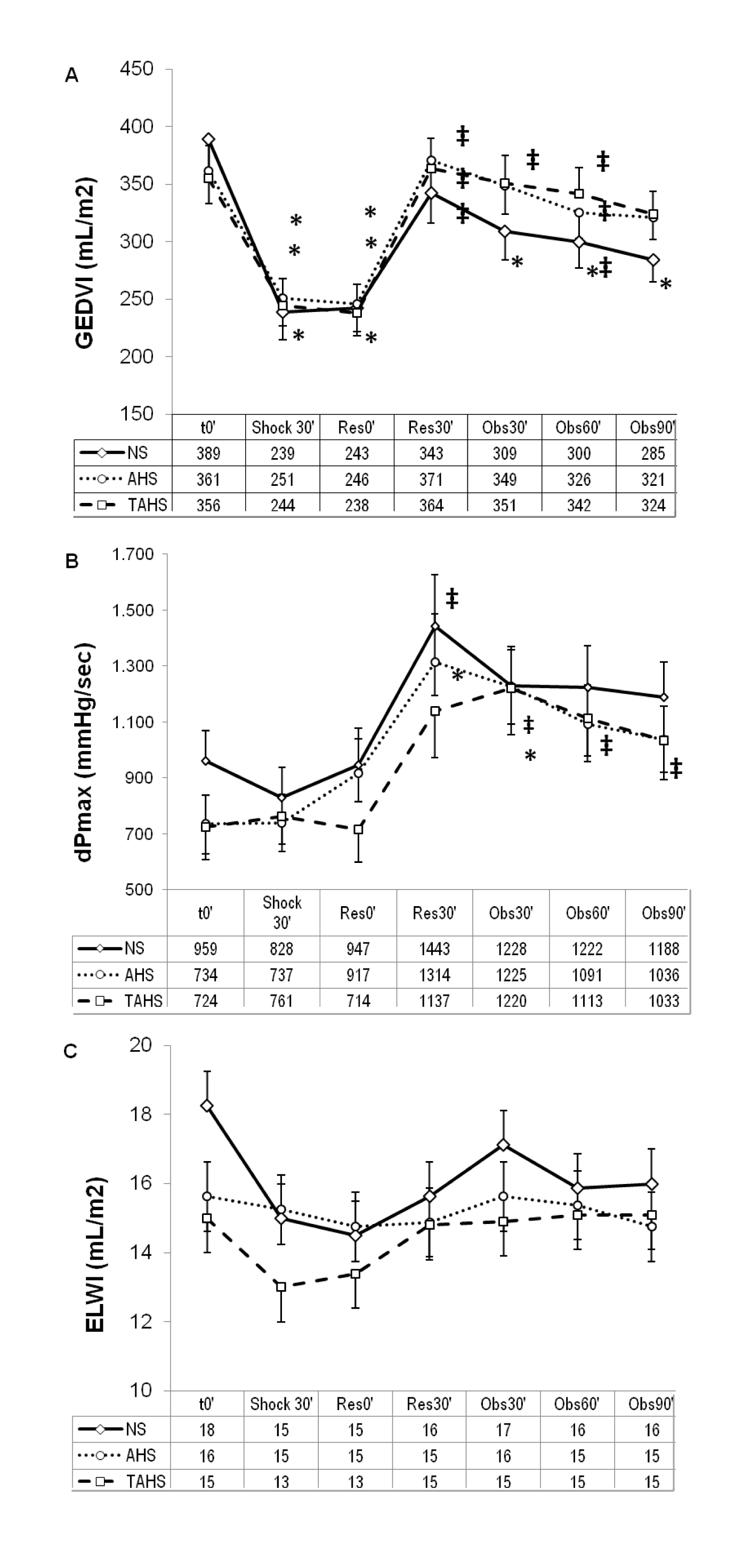

Supplement: S1 Fig — (*) Significant difference (p < 0.05) from baseline, same group. (‡) p < 0.05 from hemorrhage, same group. (#) p < 0.05 from group NS. (TIF) [file pone.0121678.s001.tif]

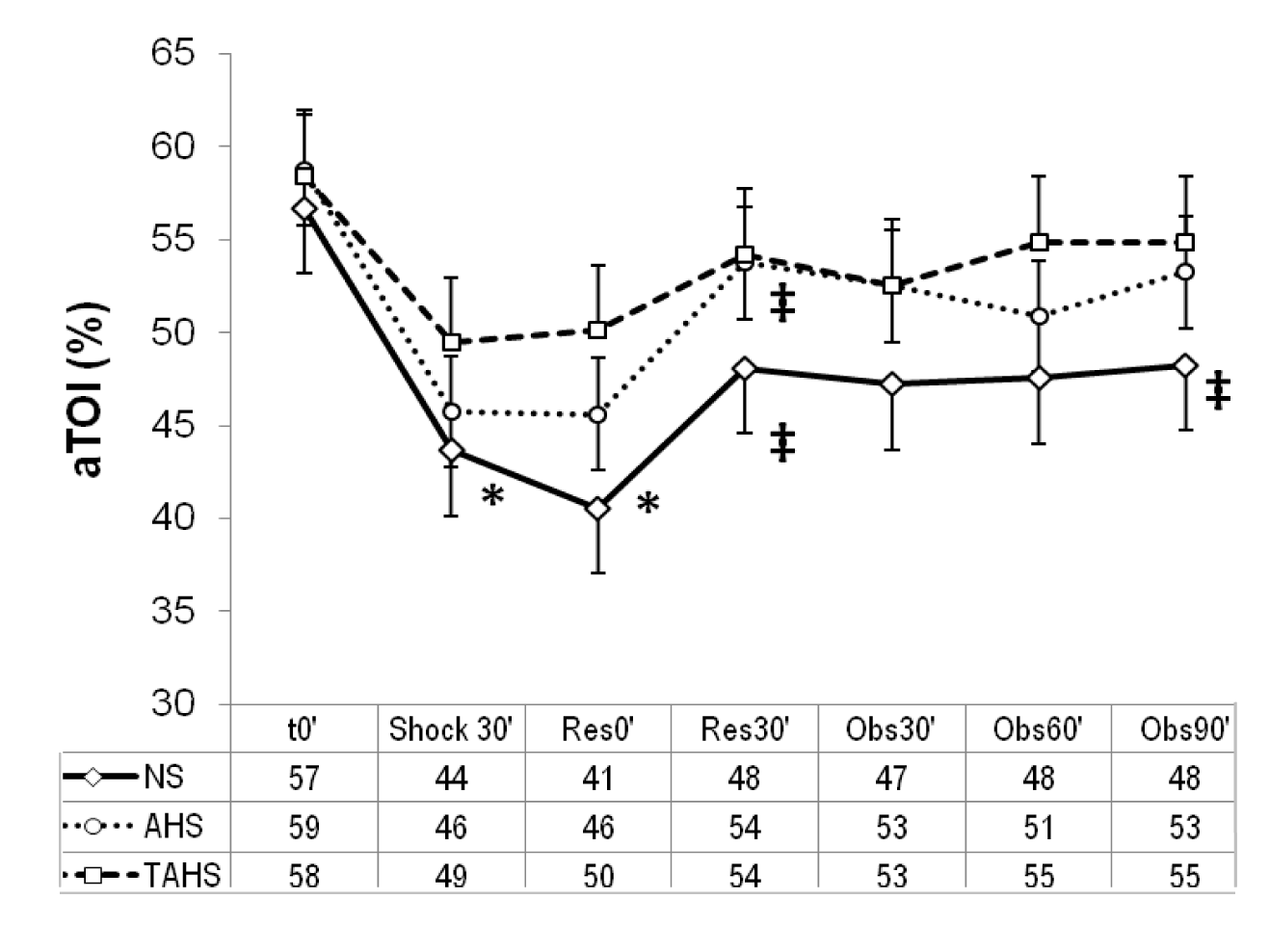

Supplement: S2 Fig — (*) Significant difference (p < 0.05) from baseline, same group. (‡) p < 0.05 from hemorrhage, same group. (#) p < 0.05 from group NS. (TIF) [file pone.0121678.s002.tif]
